# Gut Microbiota Dysbiosis Associated with Bile Acid Metabolism in Neonatal Cholestasis Disease

**DOI:** 10.1038/s41598-020-64728-4

**Published:** 2020-05-06

**Authors:** Meng Li, Sixiang Liu, Mingying Wang, Hongwei Hu, Jianwen Yin, Chuanfa Liu, Yongkun Huang

**Affiliations:** 1 Department of Pediatrics, the First Affiliated Hospital of Kunming Medical University and Yunnan Key Laboratory of Laboratory Medicine, 650032 Kunming, China; 2 Department of Gastroenterology, Children’s Hospital of Kunming Medical University, 650034 Kunming, China; 3 Yunnan Center for Disease Control and Prevention, 650100 Kunming, China; 4 Kunming Institute of Zoology, Chinese Academy of Sciences, 650000 Kunming, China; 5 University of Chinese Academy of Sciences, 100049 Beijing, China

**Keywords:** Diagnostic markers, Cholestasis

## Abstract

Neonatal cholestasis disease (NCD) is a complex and easily mis-diagnosed condition. We analyzed microbiota community structure in feces and measured short-chain fatty acids, bile acids (BAs) and liver function of 12 healthy, 13 NCD, and 13 treated infants after diagnosis. Based on 16S rRNA gene amplicon sequencing and gas-chromatographic-mass-spectrometric analysis of secondary BAs, we identified microbial genera and metabolites that associate with abnormal bile secretion. *Streptococcus gallolyticus* and *Parabacteroides distasonis*, and *Lactobacillus gasseri* had higher relative abundance in healthy and NCD infants respectively. Compared to NCD patients, healthy infants had higher LCA, CDCA and GCDCA fecal concentrations. The three microbial species and three secondary bile acids were selected as potential non-invasive combined biomarkers to diagnose NCD. We propose that microbiota-metabolite combined biomarkers could be used for diagnosis of NCD, and this may contribute to improved early clinical diagnosis of NCD in the future.

## Introduction

Neonatal cholestasis disease (NCD) affects approximately 1 in every 2500 term infants and is infrequently recognized by primary providers in the setting of physiologic jaundice. Cholestasis jaundice is mostly due to biliary atresia and frequently results from non-biliary atresia^1^. The etiology of biliary atresia is unclear but is thought to involve bile duct dysmorphogenesis, viral infection, toxins, chronic inflammation, or autoimmune-mediated bile duct injury^2–5^. Non-biliary atresia etiology of neonatal cholestasis may involve bacterial sepsis, galactosemia, tyrosinemia, panhypopituitarism, defective BA synthesis, or obstructive gallstones^1^. The complex causes for NCD necessitate improved clinical practice guidelines for the care of infants with cholestasis. Hence, joint general recommendations of the North American Society for Pediatric Gastroenterology, Hepatology, and Nutrition and the European Society for Pediatric Gastroenterology, Hepatology, and Nutrition are available for evaluation of NCD in infants, which identify: 1) the measurement of total and conjugated (direct) serum bilirubin for babies at 2 weeks of age; 2) physical examination for hepatomegaly, splenomegaly and appearance of illness; 3) direct visualization of stool pigment; 4) intra-operative cholangiogram and histological examination of the duct remnant^1^. However, infants with biliary atresia usually appear healthy and grow normally, which may deceive the parent or physician into believing that the jaundice is physiologic or caused by breastfeeding^6^. Thus, development of new or additional biomarkers is considered important in order to improve the care of NCD.

Among the most studied causes of NCD in recent years are metabolic diseases and disorders of bile transport and BA synthesis. Cumulative evidence suggests that the composition and function of the gut microbiota plays a prominent role in the occurrence of human metabolic diseases, including type II diabetes^7^, obesity^8^, liver disease^9,10^, atherosclerosis^11^, hypertension^12^, and cholelithiasis^13,14^. Notably, fecal microbiota transplantation has been successfully used in treatment of recurrent *Clostridium difficile* infection^15^. Analysis of gut microbes is proposed to have potential in diagnosis of many diseases, such as colorectal cancer (CRC)^16,17^. Importantly, gut microbiota also plays a vital role in modulating BA metabolism and host health^18^. For example, the gut microbiota is able to regulate BA metabolism by reducing levels of tauro-beta-muricholic acid, a naturally occurring farnesoid X receptor (FXR) antagonist^19^. Precision microbiome reconstitution can also restore BA-mediated resistance to *Clostridium difficile*^20^. Conversely, secondary BAs, produced solely by intestinal bacteria, can accumulate to high levels in the enterohepatic circulation of some individuals and may contribute to the pathogenesis of colon cancer, gallstones, and other gastrointestinal diseases^21^. A study by Guo *et al*. (2018) revealed altered gut microbial composition and co-occurrence of certain species in cholestatic infants compared with healthy infants^22^, indicating a metabolic disorder modulated by gut microbiota associated with occurrence of cholestatic jaundice. Zhou *et al*. (2019) reported an alteration of gut microbiota in neonatal cholestasis patients, mainly manifested as a significant increase in species richness and an increased abundance of potentially pathogenic species. The primary manifestation in jaundice patients was a significant decrease in *Bifidobacterium* (possibly involved in metabolism of bilirubin through the galactose metabolic pathway), suggesting substantial divergence of gut microbiota between neonatal cholestasis and breast milk jaundice^23^. Therefore, combined with analysis of metabolites in both serum and feces, it should be feasible to develop gut microbial markers that improve the diagnosis of cholestatic liver disease in infants.

Here, 12 healthy controls (HC) and 13 untreated neonatal cholestasis participants (NNC) were recruited for study. According to their remission status after 7 days treatment, the 13 NNC patients were divided into 8 non-remission neonatal cholestasis participants after treatment (TNCN) and 5 remission neonatal cholestasis participants after treatment (TNCR) groups. Fecal samples were obtained from all subjects for 16S rRNA-based gut microbe analysis as well as quantitative measurements of both short-chain fatty acids (SCFAs) and BAs. Serum samples from all patients were used to measure the change of common liver function indexes before and after treatment (additional file 6). Our aim was to identify gut microbial markers co-occurring with specific metabolites associated with neonatal cholestasis.

## Results

### Altered BAs in stool, liver functional indexes, and SCFAs

**Figure 1:**
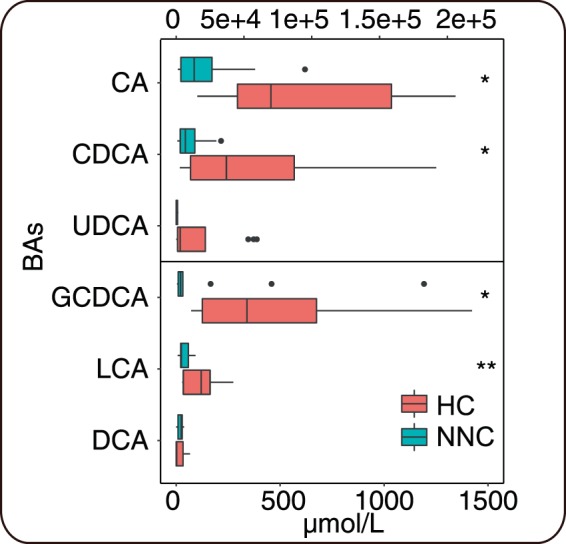
Fecal BAs concentration in healthy control (HC) group and non-treated neonatal cholestasis (NNC) group. * and ** represent p-value lower than 0.05 and 0.01 respectively.

To discover metabolic features associated with occurrence of neonatal cholestasis, we measured fecal BAs in all four groups and liver function in NNC, TNCR, and TNCN groups (Fig. 1 and additional File 1). After treatment for one week, NCD infants entered remission in the TNCR group but worsened in the TNCN group. BAs in stool, including GCDCA (*P* = 0.049), CDCA (*P* = 0.025), CA (*P* = 0.019) and LCA (*P* = 0.007), showed significantly higher concentrations in the HC group compared to the NNC group (Fig. 1). However, neither the HC and NNC groups, nor TNCR and TNCN groups, showed a significant difference in DCA and UDCA concentrations in paired t-test analysis (additional File 1,B). Comparing liver function test results in the NNC group with those of the TNCR and TNCN groups, we found that DBil, TBil, AST, and ALT were significantly lower (*P* < 0.05) in the TNCR group (additional File 1 C). No significant difference was found between the NNC and TNCN groups (additional File 1D). Moreover, the HC group had higher acetic acid concentrations than the NNC group (*P* < 0.01); however, no significant differences were found in other SCFAs (additional File 1E).

### Gut microbiota structure

**Figure 2:**
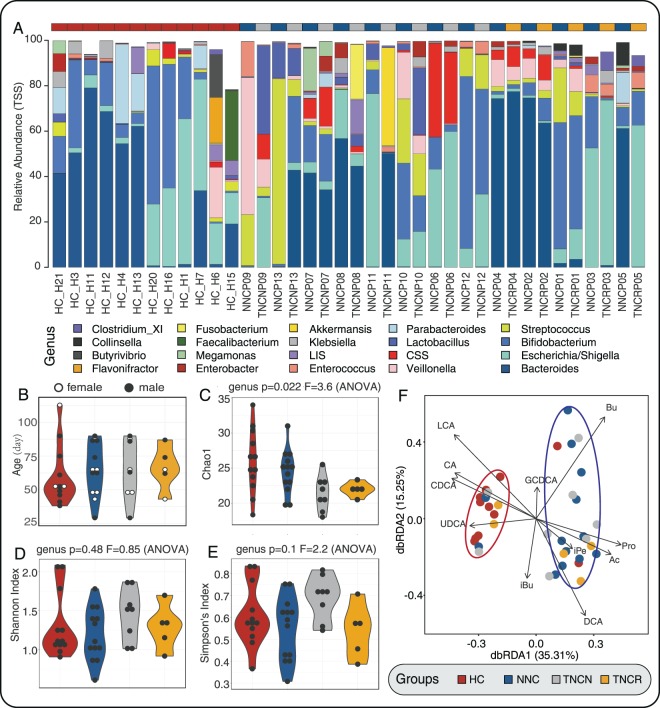
The variation of microbiota component in four groups (healthy controls (HC), non-treated neonatal cholestasis participants (NNC), non-remission neonatal cholestasis participants after treated (TNCN) and remission neonatal cholestasis participants after treated (TNCR)) and samples distribution of age and gender. (**A**) Relative abundance of microbiota in genus level (top 20 genera); (**B**) Age and gender distribution of participants (black point and blank point represent female and male neonates); (**C**–**E**) Microbiota alpha diversity (chao1, Shannon index, and Simpson’s index) of four groups; (**F**) Distance-based redundancy analysis (DB-RDA) of four groups microbiota constrained by metabolites. Colors of points, violins, and diamonds represent different groups, maroon, navy, grey, and orange represent HC, NNC, TNCN, TNCR respectively.

We obtained 1,116,189 high-quality reads from 38 fecal samples with 22,779 ± 5,984 (mean ± sd.) reads per sample. We then identified 161 OTUs in the four groups, based on 97% sequence similarity (125 OTUs in HC, 140 OTUs in NNC, 103 OTUs in TNCR and 106 OTUs in TNCN). We identified 7 phyla and 65 genera in total, assigned by RDP Classifier, including the four predominant bacterial phyla (Firmicutes, Proteobacteria, Bacteroidetes, and Actinobacteria; additional File 2A) and 10 prominent bacterial genera (*Bacteroides*, *Escherichia*/*Shigella*, *Bifidobacteriaceae*, *Streptococcus*, *Veillonella*, *Clostridium sensu stricto* (*C. sensu stricto*), *Lactobacillus*, *Enterococcus*, *Lachnospiracea incertae sedis* (*L. incertae sedis*), and *Klebsiella*; Fig. 2A). The relative abundance of gut species in HC and NNC groups showed significant differences at the genus level - *Alloprevotella, Barnesiella, Collinsella, Dialister, Enterobacter, Megamonas, Megasphaera, Senegalimassilia, Staphylococcus, Tannerella, and Veillonella* were enriched in NNC groups; *Blautia*, *Butyrivibrio*, *Clostridium_XI*, *Faecalibacterium*, *Flavonifractor*, *Halomonas*, *Holdemanella*, *Parabacteroides*, *Pelagibacterium*, *Roseburia*, and *Sutterella* were enriched in the HC group (additional File 3). Moreover, NNC and TNCN samples were enriched in *Alloprevotella, Clostridium_XlVa and Tannerella*, and *Aggregatibacter*, respectively. Compared to the TNCR group, the NNC group was enriched in *Tannerella* (additional File 3). Both HC and NNC groups presented with higher microbial richness (Chao1, *P* = 0.022; richness, *P* = 0.019) and diversity (Shannon index, *P* = 0.48; Simpson index, *P* = 0.1; Fig. 2C–E, additional File 4 A and B). According to beta diversity analysis, the four groups had miscellaneous microbiota compositions and large microbiota variation and did not cluster distinctly into different groups (non-metric multi-dimensional scaling (NMDS) stress = 0.22 and principal coordinates analysis (PCoA); additional File 4 C and D). Distance-based redundancy analysis (dbRDA) showed that HC and TNCR (red circle), along with TNCN and NNC (blue circle), had similar fecal microbiota compositions. Physiological factors, such as LCA, CA, CDCA, and UDCA, correlated to the microbiota communities in HC and TNCR groups. Correspondingly, Bu, Pro, Ac, iPe, and DCA correlated to gut microbiota in NNC and TNCN groups (Fig. 2F).

### Selection of important features related to NCD

**Figure 3:**
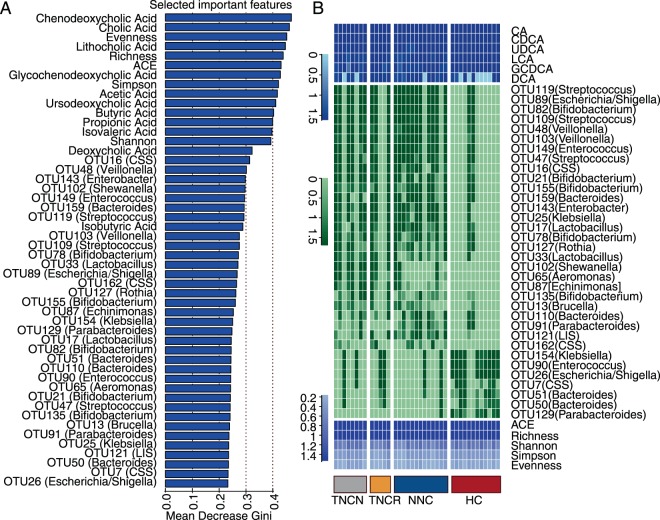
Selected important features and the differences of features in the genus level. (**A**) The top 50 important features of participation were shown according to the Mean Decrease Gini. (**B**) the relative abundance of microbiota (green), the concentration of BAs in the feces (deep blue), and microbiota diversity indexes (purple) were shown after a logarithmic transformation.

To identify microbial or metabolic features related to neonatal cholestasis, we applied the random forest algorithm to identify factors that may promote specific gut microbial species, between NCD and healthy babies. As shown in Fig. 3A, 50 factors (Mean Decrease Gini index > 0.2), including BAs, SCFAs, microbiota at OTU level and diversity indexes, were the most important features characteristic of neonatal cholestasis. From these 50 factors, 6 BAs, 5 SCFAs and 34 OTUs were selected as important affected features. Genera including *Bifidobacteriaceae*, *Bacteroides*, *C. sensu stricto*, *Streptococcus*, *Lactobacillus*, *Klebsiella*, *Escherichia/Shigella*, *Enterococcus*, *Parabacteroides*, and *Veillonella*, that may contribute to primary BA metabolism, were selected. Figure 3B shows the corresponding microbial abundance and bile acid indexes - TNCN and NNC, as well as TNCR and HC groups, had similar microbiota compositions. HC and NNCR groups had higher abundance of *Bacteroides*, *Klebsiella*, *Enterococcus*, *Escherichia*/*Shigella*, and *Parabactrioides*. However, TNCN and NNC groups were mainly enriched in *Bifidobacteriaceae*, *Lactobacillus*, *Streptococcus*, *Veillonella*, and *C. sensu stricto* (Fig. 3B). All the features found above were significant potential biomarkers.

### Correlation of microbial species with BAs

**Figure 4:**
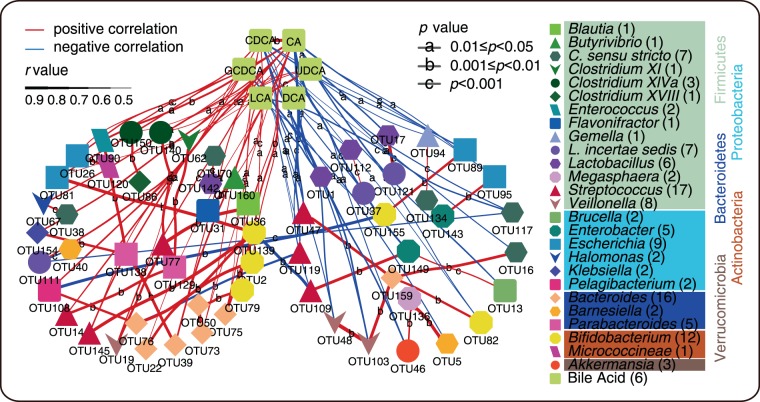
Significant correlative relation between BAs and microbiota. Colorful and various shapes points in the network mean OTUs or BAs, the color of points means genus of OTUs (numbers in the bracket present OTU numbers). The colorful panes under the genus names mean different phyla (phylum name own the same color with the panes’). The red and blue lines mean positive and negative correlation respectively. The width of lines and letters on the line represents the *r*-value and the *p*-value of the correlative relation respectively.

In order to confirm the results, we determined correlations between gut microbes and BAs, which were represented by three circles. The results showed that species quantities were associated with BAs, especially for *Streptococcus*, *Bacteroides*, *Bifidobacterium*, *Escherichia*, *Veillonella*, *C. sensu stricto*, *Lactobacillus*, *Enterobacter*, and *Parabacterioides* genera. Most of these microbes showed significant differences with CDCA and CA; a subset of species showed a positive or negative correlation with DCA, GCDCA, and LCA (Fig. 4). As shown in Fig. 4, *Bacteroides*, *Pelagibacterium*, *L. incertae sedis*, *Klebsiella*, *Halomonas*, *Enterococcus* and *Clostridium XlVa* had a positive correlation with BAs. However, *Lactobacillus*, *C. sensu stricto*, *Barnesiella* and *Akkermansia* had a negative correlation with BAs.

### Functional analysis of microbiota

**Figure 5:**
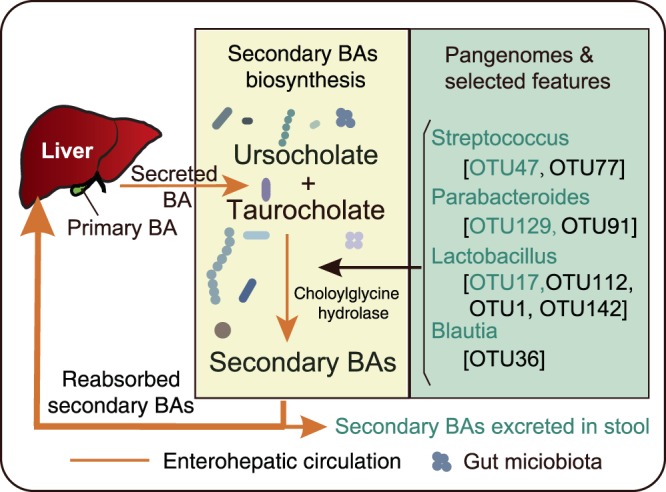
Potential Biomarkers action mechanism. Pale yellow and laurel-green panes present secondary BAs biosynthesis in the intestinal environment and selected features of microbiota respectively. The yellow line means enterohepatic circulation, and vesicles mean gut microbiota. Green texts in the laurel-green panes represent selected by both feature selection analysis and correlation statistical analysis, and the black texts found by a single analysis.

A total of 72 OTUs representing potential microbiota biomarkers were selected from our important features and correlation analyses. We then used representative 16S rRNA gene sequences of the 72 OTUs to screen against the Microbial Whole-Genome Database in NCBI. We obtained 72 best hits that matched to previously sequenced complete genomes of microbial species (additional File 5). We submitted the 72 matched genomes to KEGG and mapped them to secondary BA biosynthesis pathways (ko00121). We found 9 of the 72 whole-genome-known microbes possessed choloylglycine hydrolase [EC:3.5.1.24], which contributes to primary BA metabolism. These 9 microbes mainly belonged to the *Streptococcus*, *Parabacteroides*, *Lactobacillus* and *Blautia* genera (additional File 5). We found that these target gut microbes possess choloylglycine hydrolase genes, possibly contributing to BA metabolism (Fig. 5). According to the above analysis, we identified possible essential biomarkers in *Streptococcus* (OTU47, OTU77), *Parabacteroides* (OTU129, OTU91), *Lactobacillus* (OTU1, OTU112, OTU142, OTU17), and *Blautia* (OTU36) genera. Within these, *Streptococcus gallolyticus* subsp. *Gallolyticus* (*S. gallolyticus*) (OTU77), *Parabacteroides distasonis* (*P. distasonis*) (OTU129) and *Lactobacillus gasseri* (*L. gasseri*) (OTU17), identified in both correlation and important features analysis, were the best potential biomarkers for diagnosis.

## Discussion

Currently, NCD is one of the most frequent mortality risks for neonates, owing to challenges in its accurate and rapid diagnosis^1^. Clinicians typically use a liver function test, B-ultrasonography examination, stool color chart test, and even an invasive contrast examination approach, to diagnose NCD in infants. These tests may result in a “BA black box” effect, making it difficult to ascertain the real status of the biliary tract accurately, since liver function tests and stool color charts only reflect the beginning and end of biliation. Thus, with current clinical diagnosis, we hardly know the patency situation of biliary tract. Moreover, biliary tract B-type ultrasonography examinations have limited imaging techniques which judge the biliary tract patency clearly^24^. Furthermore, contrast examination or biliary cannulation introduces infection risks and difficulty in acceptance by patients or legal guardians. New and non-invasive diagnostic approaches are needed for routine diagnosis of NCD. Interestingly, researchers have found that microbiota and microbiota-sourced metabolites may present valuable potential biomarkers for disease diagnosis, including autism^25^, irritable bowel syndrome^26^, pediatric necrotizing enterocolitis^27^, inflammatory bowel disease^28,29^, CRC^30–32^, atherosclerosis^33^ and Hashimoto’s thyroiditis^34^. Similarly, research has revealed that the gut microbiota participates in the regulation of primary BA metabolism^18^ and contributes to both enterohepatic circulation and biliary tract health^14,35^. Furthermore, the microbiota could affect the occurrence and development of liver diseases^36^. Investigators have also used a random-forest model and function prediction to correlate microbiota with cholestasis of infants^37^. In our research, we used both the random forest model and correlation analyses to search for potential microbial biomarkers and combined this with intestinal metabolites to find candidates that may participate in secondary BA biosynthesis. Two mutually supporting analyses, described here, demonstrate superior reliability and avoidance of false positives.

Although the gut microbiota structure of infants showed large variations, possibly caused by gender, living environment or genetic background^38^, we found that NCD infants have a higher abundance of *Streptococcus* and *Lactobacillus*. Meanwhile, in the healthy group and remission patients, these two microbes were decreased. *S. gallolyticus*, *P. distasonis* and *L. gasseri* were the best potential biomarkers we discovered. *S. gallolyticus* is known to be a species associated with colorectal cancer, activating the Wnt signaling pathway and leading to downregulation of the BA transporter Slc10A2, resulting in accumulation of BAs^39^. Therefore, *S. gallolyticus* may be an underlying factor causing NCD. *L. gasseri* has been reported to regulate whole-body glucose homeostasis in the upper small intestine^40^, but the mechanism linking *L. gasseri* to NCD is unclear. *P. distasonis* is known for its function of decreasing weight gain, hyperglycemia and hepatic steatosis via regulation of BA metabolism^41^. During metabolism of BAs, primary BAs are conjugated to substrates, secreted into the bile and stored in the gallbladder, then excreted into the intestine^42^. BAs are then de-conjugated to secondary BAs under the influence of nuclear receptor FXR and bile salt hydration enzymes^18^. In our study, primary BAs (CA and CDCA) in the NNC group were lower than in the healthy group, revealing that NNC patients could not excrete primary BAs like healthy babies. BSH is a member of the choloylglycine hydrolase family and can de-conjugate tauro- or glycol-conjugated BAs^43^. Two genera carry choloylglycine hydrolase genes, this being one of the most critical enzymes for primary BA metabolism to the secondary BAs^18,21^. Researchers have also reported that *Streptococcus* and *Lactobacillus* are closely associated with primary BA metabolism and secondary BA biosynthesis in the intestinal tract, due to the bile metabolism-related enzymes they express^18,37,44^. Associations have also been found between secondary BAs and metabolic diseases, including hepatic function^19,37^, type II diabetes and inflammatory bowel disease^42^. The concentrations of GCDCA and LCA, two secondary BAs, in NCD babies’ feces were significantly lower than those in healthy babies in this study. Cholestasis or congenital biliary atresia affects primary BA excretion as well as biosynthesis of secondary BAs, so our result reveals that secondary BAs in feces could be valuable potential biomarkers in the clinic. Acetic acid in stools showed significant differences in our study, but other SCFAs did not - this may be caused by similar breastfeeding patterns and similar abundance of SCFA-producing microbes (mainly the *Ruminococcaceae* and *Lachnospiraceae* families). Although our results reveal reliable associations between our potential biomarkers and NCD, more clinical verification and evidence are needed to confirm the future utility of this approach with regard to exceptional circumstances such as inborn errors of BA metabolism.

In conclusion, we propose a potential combined-biomarker diagnostic method, consisting of microbes *S. gallolyticus, P. distasonis and L. gasseri*, alongside LCA and GCDCA. As a non-invasive diagnostic method, this may decrease the mis-diagnosis rate and avoid the discomfort of invasive methods in infants, subject to clear clinical validation. Our conclusion requires support from animal model data and further clinical evidence. The approach of combining gut microbiota with metabolite data could however provide a universal and reliable method to diagnose NCD. It could be employed earlier, being non-invasive, and made available to the weakest and youngest patients, increasing future take-up of the technology.

## Material and Methods

### Participant enrollment and sample collection

Infants with cholestasis jaundice were enrolled from inpatients in the Department of Pediatrics, First Affiliated Hospital of Kunming Medical University, and from inpatients of the Department of Gastroenterology, Affiliated Children’s Hospital of Kunming Medical University. They included 7 males and 6 females, age 65.2 ± 19.4 days (Fig. 2B). All legal guardians of participants gave their written informed consent before the start of the study, which was conducted under Kunming Medical University ethics committee approval and guidance. All methods were performed in accordance with the committee guidelines and regulations. All of the babies satisfied the following criteria: 1) Levels of serum direct bilirubin were higher than 1.0 mg/dl (17 mmol/l), or levels of gamma-glutamyl transpeptidase (GGT) were higher than 10 mmol/l; 2) Age 28 to 120 days; 3) All infants were mixed fed without supplementary food; 4) No use of antibiotics or probiotics for 2 weeks before the study; 5) No diarrhea. The first fecal sample from each patient was collected before treatment. After the first collection of fecal samples, all patients were given hepatoprotective and choleretic treatments: ursodeoxycholic acid, 5 mg/dose, taken orally three times a day and reduced glutathione, 0.3 g/dose, once daily by intravenous drip. The second fecal specimen was collected at least seven days after treatment - the post-treatment group. We made a follow-up visit of all NCD infants (Additional file 6) in July 2019. All remission infants and five out of eight non-remission infants were cured finally, one non-remission infant died after transferred to another hospital for 7 months, and two were lost to follow-up.

Healthy infants (HC, healthy controls) were selected from the Preventive Health Department of the First Affiliated Hospital of Kunming Medical University, from November 2014 to November 2015. 12 healthy infants were enrolled, including 8 males and 4 females, age 60.2 ± 22.0 days, who met the following criteria: 1) Age 28 to 120 days; 2) Mixed fed without supplementary food; 3) No antibiotics or probiotics for 2 weeks before study; and 4) No diarrhea. Fecal samples from healthy controls were collected during physical examination, but no blood samples were taken due to ethical considerations.

Fresh feces was collected into sterile fecal collection boxes and transported on dry ice before storage at −80 °C prior to further analysis. Blood samples were collected during hospitalization for immediate liver function tests.

### Measurement of fecal short-chain fatty acids

About 200 mg of frozen feces was thawed on ice and homogenized by adding 1 ml phosphoric acid (0.5%) saturated with sodium chloride. 2 ml of ether was then added, and the mixture shaken intermittently. Mixed samples were centrifuged at 4000 rpm for 10 min, and the supernatants collected and filtered through 0.22 μm syringe filters prior to detection of short-chain fatty acids (SCFAs; including acetic acid, propionic acid, butyric acid, iso-butyric acid, and iso-pentoic acid).

SCFAs were quantitatively measured with a gas-chromatogram (456-CG; Varian) equipped with flame ionization detector and KB624 (30 m×0.32 mm×1.8 um) chromatographic column. Highly purified nitrogen (N_2_) was used as the carrier gas. The initial column temperature was 50 °C sustained for 5 minutes; rising by 10 °C/min until it reached 150 °C, sustained for 10 minutes; then rising at 20 °C/min to 200 °C, sustained for 12 minutes. The temperatures of the injector and detector were set at 230 and 280 °C respectively.

### Liver function tests and fecal BA Measurements

Fresh blood samples were incubated at 37 °C until coagulated, then centrifuged at 3000 rpm for 10 minutes, with supernatants being stored at −20 °C. Liver function tests, including alanine aminotransferase (ALT), aspartate aminotransferase (AST), total protein (TP), albumin (ALB), total bilirubin (TBil), indirect bilirubin (IBil), gamma-glutamyl transpeptidase (GGT), total serum bile acid (TBA) and alkaline phosphatase (ALP) indexes in serum, were measured by Hitachi-7100 automatic biochemical analyzer (Hitachi, Ltd., Japan).

About 200 mg of frozen feces was placed in a 10 ml tube and thawed at room temperature. Two ml of methanol was added to the tube, which was shaken and mixed intermittently. Mixed samples were centrifuged for 5 min at 4000 rpm. The supernatants were collected, filtered through 0.22 μm syringe filters, and stored in brown sample bottles for later detection of BAs.

Liquid chromatography-mass spectroscopy (LC/MS) was used to detect BA concentrations in feces. Analysis of BAs was performed using Agilent 1290 Infinity HPLC, with a ZORBAX RRHD column (2.1 mm × 50 mm × 1.8 μm). The mobile phase A was 0.1% formic acid-water, and phase B was acetonitrile. The flow rate was 300 μl/min during the entire procedure. Gradient elution started with 95% A from 0 to 5 minutes, then decreased to 60% A from 5 to 15 minutes and to 5% A from 15 to 15.2 minutes, which was maintained up to 17 minutes. After that, 5% A increased to 95% from 17 to 17.2 minutes and maintained to 19 minutes. B was changed according to the percentage of A in the solution. The retention times of glycochenodeoxycholic acid (GCDCA), ursodeoxycholic acid (UDCA), chenodeoxycholic acid (CDCA), deoxycholic acid (DCA), CA (cholic acid), and lithocholic acid (LCA) were 13.41, 11.71, 13.04, 13.30, 12.00, and 14.57 minutes respectively. The AB Sciex API 4000^TM^ LC/MS/MS system was used to detected these components. The precursor ions of GCDCA, UDCA, CDCA, DCA, CA, and LCA were recorded at 448.3, 391.2, 391.2, 391.2, 407.2, and 375.3 m/z, respectively; collision energy was set at −77, −30, −30, −30, −30 and −40 V, respectively; entrance potential was 10 V and collision cell exit potentials were −11, −20, −20, −20, −20 and −20 V, respectively.

### Illumina sequencing of bacterial 16S rRNA

About 200 mg of feces per sample was used to extract fecal DNA using the Qiagen QIAamp DNA Stool Mini Kit following the manufacturer’s instructions. DNA concentration of samples was determined fluorometrically and all samples were normalized to 5.00 ng/μl for PCR amplification. Bacterial 16 S rRNA amplicons were generated via amplification of the V3-V4 hypervariable region of the 16 S rRNA gene using a common primer pair 341 F (CCTACGGGNGGCWGCAG) / 785 R (GACTAC HVGGGTATCTAATCC) and the following parameters: 94 °C^(3:00)^ + [94 °C^(0:10)^+ 55 °C^(0:15)^+72 °C^(0:30)^] × 20 cycles +72 °C^(7:00)^. Amplicons were then pooled for sequencing using the Illumina MiSeq platform with 2×250 bp paired-end reads. Samples returning greater than 10,000 reads were deemed to have successful amplification.

### Bioinformatics and statistical methods

Sequence reads with quality scores less than 20 at any one site were filtered out. Additionally, any reads less than 400 bp were removed. De-replication, chimera filtration, and Operational Taxonomic Unit (OTU) clustering of the remaining reads were analyzed using USEARCH (version 11), with reference to the USEARCH manual (https://www. drive5.com/usearch/manual/). Briefly, OTUs were clustered according to 97% sequence similarity using the UPARSE algorithm^45^ and the taxonomic units were assigned to each OTU using RDP Classifier (version 16). The sequence abundance of each OTU within each sample was calculated by counting reads that mapped to the representative sequence of each OTU. OTUs present in at least two samples with at least six sequence reads in each sample were used in further analyses.

The Random Forest Algorithm was used to select essential microbes and metabolic features associated with neonatal cholestasis disease. Filtering factors included microbial species, SCFAs and BAs in stool, liver function test indexes and microbial diversity indexes. We selected 31 samples (~80% of the total) as the training sets and 7 samples (~20% of the total) as test sets in this classifier. The top 50 most important features were selected and expressed as the Mean Decrease Gini index exported by Random Forest analysis. We also constructed a co-occurrence network to find potential associations between gut microbiota and BAs and calculated Spearman’s rank correlation coefficients between microbiota and BAs. Co-occurring pairs, with Spearman’s rank correlation coefficient |r | > 0.5, were selected for further analysis. Microbes directly correlated with BAs with the highest correlation coefficients were defined as dominant microbes involved in BA metabolism. Amounts of microbes were considered as being indirectly correlated to BAs or dominant microbes. Only the two types of microbes mentioned above are shown in our network (Fig. 4). The correlation network was visualized by Cytoscape (version 3.7.0).

To demonstrate the biological significance of the target microbes identified by the two methods above, we first compared target microbes with the known microbiota database by screening their representative sequences against the 16 S ribosomal RNA sequence (Bacteria and Archaea) database on NCBI. Then we predicted their functions by identifying whether target microbes had key enzymes involved in BA metabolism, using the Pangenome database in the Kyoto Encyclopedia of Genes and Genomes (KEGG) (https://www.genome.jp/kegg/genome.html). We used “Secondary BA biosynthesis” (pathway id: ko00121) as a reference pathway. Seven key enzymes contribute to the metabolism of primary BAs (mainly CA and CDCA) to secondary BAs (mainly DCA, LCA and UCDA), including choloylglycine hydrolase [EC:3.5.1.24], 7-alpha-hydroxysteroid dehydrogenase [EC:1.1.1.159], 7- beta-hydroxysteroid dehydrogenase (NADP^+^) [EC:1.1.1.201], 3-alpha-hydroxycholanate dehydrogenase (NAD^+^) [EC: 1.1.1.52], 3-alpha-hydroxycholanate dehydrogenase (NADP^+^) [EC:1.1.1.392], 3-beta- hydroxycholanate-3-dehydrogenase (NAD^+^) [EC:1.1.1.391] and 3-beta-hydroxycholanate-3-dehydrogenase (NADP^+^) [EC:1.1.1.393]. Microbial species with at least one of the seven enzymes were considered to participate in bile acid metabolism.

Multiple comparison tests were performed by paired-sample Wilcoxon signed-rank tests using R and ANOVA on Graph-pad prism 7 for Windows (Graphpad software, lnc., USA). We used the Benjamini and Hochberg procedure to correct p-values for multiple comparisons. Statistical significance was set at *P* < 0.05.

### Ethics Approval And Consent To Participate

This study was approved by the Medicine Ethics Committee of Kunming Medical University. All the infants’ parents voluntarily allowed their children to participate in the investigation of scientific research.

## Supplementary information

Additional file 1-4.

Additional file 5 and 6.

## Data Availability

All raw sequence data generated in this research has been deposited in the Genome Sequence Archive (GSA)^46^ in BIG Data Center^47^, Beijing Institute of Genomics, Chinese Academy of Sciences, under accession number CRA001920 and is publicly accessible at https://bigd.big.ac.cn/gsa.

## References

[1] Fawaz R (2017). Guideline for the Evaluation of Cholestatic Jaundice in Infants: Joint Recommendations of the North American Society for Pediatric Gastroenterology, Hepatology, and Nutrition and the European Society for Pediatric Gastroenterology, Hepatology, and Nutrition. Journal of pediatric gastroenterology and nutrition.

[2] Sokol RJ, Mack C (2001). Etiopathogenesis of biliary atresia. Semin Liver Dis.

[3] Mack CL (2007). The pathogenesis of biliary atresia: evidence for a virus-induced autoimmune disease. Semin Liver Dis.

[4] Schreiber RA, Kleinman RE (1993). Genetics, immunology, and biliary atresia: an opening or a diversion?. J Pediatr Gastroenterol Nutr.

[5] Bezerra JA (2005). Potential etiologies of biliary atresia. Pediatric Transplantation.

[6] Moyer V (2004). Guideline for the evaluation of cholestatic jaundice in infants: recommendations of the North American Society for Pediatric Gastroenterology, Hepatology and Nutrition. J Pediatr Gastroenterol Nutr.

[7] Zhao L (2018). Gut bacteria selectively promoted by dietary fibers alleviate type 2 diabetes. Science.

[8] Ridaura, V. K. *et al*. Gut Microbiota from Twins Discordant for Obesity Modulate Metabolism in Mice. Science 341, 10.1126/science.1241214 (2013). 10.1126/science.1241214 PMC3829625 24009397

[9] Zhang Z (2013). Large-Scale Survey of Gut Microbiota Associated With MHE Via 16S rRNA-Based Pyrosequencing. Am J Gastroenterol.

[10] Qin, N. *et al*. Alterations of the human gut microbiome in liver cirrhosis. Nature 513, 59-64, 10.1038/nature13568, http://www.nature.com/nature/journal/v513/n7516/abs/nature13568.html#supplementary-information (2014). 10.1038/nature13568 25079328

[11] Zhu W (2016). Gut Microbial Metabolite TMAO Enhances Platelet Hyperreactivity and Thrombosis Risk. Cell.

[12] Santisteban MM (2017). Hypertension-Linked Pathophysiological Alterations in the Gut. Circulation research.

[13] Wu T (2013). Gut microbiota dysbiosis and bacterial community assembly associated with cholesterol gallstones in large-scale study. BMC Genomics.

[14] Keren N (2015). Interactions between the intestinal microbiota and bile-acids in gallstones patients. Environmental Microbiology Reports.

[15] van Nood, E. *et al*. Duodenal Infusion of Donor Feces for Recurrent Clostridium difficile. New England Journal of Medicine 0, null, 10.1056/NEJMoa1205037 (2013). 10.1056/NEJMoa1205037 23323867

[16] Yu J (2015). Metagenomic analysis of faecal microbiome as a tool towards targeted non-invasive biomarkers for colorectal cancer. Gut.

[17] Tilg H, Adolph TE, Gerner RR, Moschen AR (2018). The Intestinal Microbiota in Colorectal Cancer. Cancer Cell.

[18] Wahlström A, Sayin, Sama I, Marschall H-U, Bäckhed F (2016). Intestinal Crosstalk between Bile Acids and Microbiota and Its Impact on Host Metabolism. Cell Metabolism.

[19] Sayin SamaI (2013). Gut Microbiota Regulates Bile Acid Metabolism by Reducing the Levels of Tauro-beta-muricholic Acid, a Naturally Occurring FXR Antagonist. Cell Metabolism.

[20] Buffie CG (2014). Precision microbiome reconstitution restores bile acid mediated resistance to Clostridium difficile. Nature.

[21] Ridlon JM, Kang D-J, Hylemon PB (2006). Bile salt biotransformations by human intestinal bacteria. Journal of Lipid Research.

[22] Guo, C. *et al*. Alterations of Gut Microbiota in Cholestatic Infants and Their Correlation With Hepatic Function. Frontiers in Microbiology 9, 10.3389/fmicb.2018.02682 (2018). 10.3389/fmicb.2018.02682 PMC6243132 30483228

[23] Zhou S (2019). Association of serum bilirubin in newborns affected by jaundice with gut microbiota dysbiosis. The Journal of Nutritional Biochemistry.

[24] Mittal V (2011). Role of abdominal sonography in the preoperative diagnosis of extrahepatic biliary atresia in infants younger than 90 days. AJR Am J Roentgenol.

[25] Kang DW (2018). Differences in fecal microbial metabolites and microbiota of children with autism spectrum disorders. Anaerobe.

[26] Hollister EB (2019). Leveraging Human Microbiome Features to Diagnose and Stratify Children with Irritable Bowel Syndrome. Journal of Molecular Diagnostics.

[27] Pang, T., Leach, S. T., Katz, T., Day, A. S. & Ooi, C. Y. Fecal biomarkers of intestinal health and disease in children. Frontiers in Pediatrics 2, 10.3389/fped.2014.00006 (2014). 10.3389/fped.2014.00006 PMC3904282 24479111

[28] Heinken A (2019). Systematic assessment of secondary bile acid metabolism in gut microbes reveals distinct metabolic capabilities in inflammatory bowel disease. Microbiome.

[29] De Preter V (2015). Metabolomics in the Clinical Diagnosis of Inflammatory Bowel Disease. Digestive Diseases.

[30] Liang QY (2017). Fecal Bacteria Act as Novel Biomarkers for Noninvasive Diagnosis of Colorectal Cancer. Clinical Cancer Research.

[31] Mangifesta, M. *et al*. Mucosal microbiota of intestinal polyps reveals putative biomarkers of colorectal cancer. *Scientific reports* **8**, 10.1038/s41598-018-32413-2 (2018). 10.1038/s41598-018-32413-2 PMC6143603 30228361

[32] Geng J (2014). Co-occurrence of driver and passenger bacteria in human colorectal cancer. Gut Pathog.

[33] Jonsson, A. L. & Backhed, F. Role of gut microbiota in atherosclerosis. Nat Rev Cardiol advance online publication, 10.1038/nrcardio.2016.183 (2016). 10.1038/nrcardio.2016.183 27905479

[34] Zhao FY (2018). Alterations of the Gut Microbiota in Hashimoto’s Thyroiditis Patients. Thyroid.

[35] Wu T (2013). Gut microbiota dysbiosis and bacterial community assembly associated with cholesterol gallstones in large-scale study. BMC genomics.

[36] Hartmann, P. *et al*. Modulation of the intestinal bile acid–FXR–FGF15 axis improves alcoholic liver disease in mice. Hepatology, n/a-n/a, 10.1002/hep.29676 (2017). 10.1002/hep.29676 PMC5962369 29159825

[37] Guo, C. *et al*. Alterations of Gut Microbiota in Cholestatic Infants and Their Correlation With Hepatic Function. Frontiers in microbiology 9, 10.3389/fmicb.2018.02682 (2018). 10.3389/fmicb.2018.02682 PMC6243132 30483228

[38] Yassour M (2018). Strain-Level Analysis of Mother-to-Child Bacterial Transmission during the First Few Months of Life. Cell host & microbe.

[39] Pasquereau-Kotula E, Martins M, Aymeric L, Dramsi S (2018). Significance of Streptococcus gallolyticus subsp. gallolyticus Association With Colorectal Cancer. Frontiers in microbiology.

[40] Bauer PV (2018). Lactobacillus gasseri in the Upper Small Intestine Impacts an ACSL3-Dependent Fatty Acid-Sensing Pathway Regulating Whole-Body Glucose Homeostasis. Cell Metab.

[41] Wang K (2019). Parabacteroides distasonis Alleviates Obesity and Metabolic Dysfunctions via Production of Succinate and Secondary Bile Acids. Cell Rep.

[42] Kuipers F, Bloks VW, Groen AK (2014). Beyond intestinal soap–bile acids in metabolic control. Nature reviews. Endocrinology.

[43] Chand D, Panigrahi P, Varshney N, Ramasamy S, Suresh CG (2018). Structure and function of a highly active Bile Salt Hydrolase (BSH) from Enterococcus faecalis and post-translational processing of BSH enzymes. Biochim Biophys Acta Proteins Proteom.

[44] Gu Y (2017). Analyses of gut microbiota and plasma bile acids enable stratification of patients for antidiabetic treatment. Nature communications.

[45] Edgar, R. C. UPARSE: highly accurate OTU sequences from microbial amplicon reads. Nat Meth 10, 996-998, 10.1038/nmeth.2604,http://www.nature.com/nmeth/journal/v10/n10/abs/nmeth.2604.html#supplementary-information (2013). 10.1038/nmeth.2604 23955772

[46] Wang Y (2017). GSA: Genome Sequence Archive. Genomics Proteomics Bioinformatics.

[47] Members BIGDC (2019). Database Resources of the BIG Data Center in 2019. Nucleic Acids Res.

