# Supplementary material for: Gut Microbiota Dysbiosis Associated with Bile Acid Metabolism in Neonatal Cholestasis Disease
Source: Sci Rep. 2020 May 6;10:7686. doi: 10.1038/s41598-020-64728-4 (PMC7203226; doi:10.1038/s41598-020-64728-4)
Supplement: Supplementary file 1 — Additional file 1-4. [file 41598_2020_64728_MOESM1_ESM.pdf]

Gut Microbiota Dysbiosis Associated with Bile Acid Metabolism in Neonatal Cholestasis Disease

Meng Li[1†], Sixiang Liu[2], Mingying Wang[2], Hongwei Hu[1], Jianwen Yin[3], Chuanfa Liu [4,5†*], Yongkun Huang [1*]

AUTHORS DETAILS

1 Department of Pediatrics, the First Affiliated Hospital of Kunming Medical University and Yunnan Key Laboratory of Laboratory Medicine, Kunming, China 650032

2 Department of Gastroenterology, Children's Hospital of Kunming Medical University, Kunming, China 650034

3 Yunnan center of disease control, Kunming, China 650100

4 Kunming Institute of Zoology, Chinese Academy of Sciences, Kunming, China 650000;

5 University of Chinese Academy of Sciences, Beijing, China 100049

† These authors contributed equally to this work.

* Corresponding authors: Yongkun Huang, hykkmyncnwd@163.com; Chuanfa Liu, liuchuanfa15@mails.ucas.ac.cn

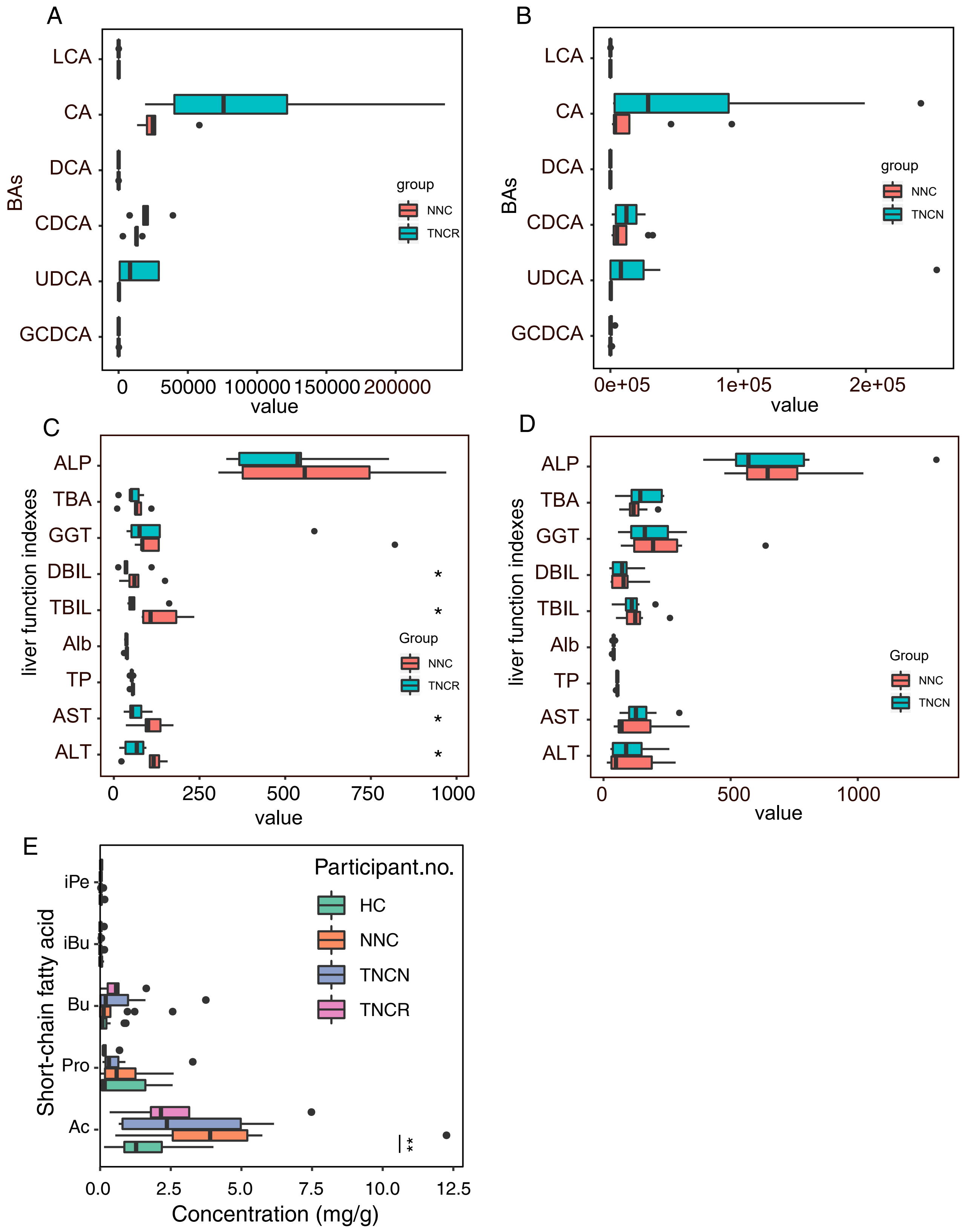

Additional file 1: BAs concentration (A and B) and liver function tests indexes(C and D) in stools results among NNC, TNCN, and TNCR groups, and SCFAs (E) results in four groups. Indexes unites of A, B, C and D were shown in additional file 6. *, *P*<0.05; **, *P*<0.01.

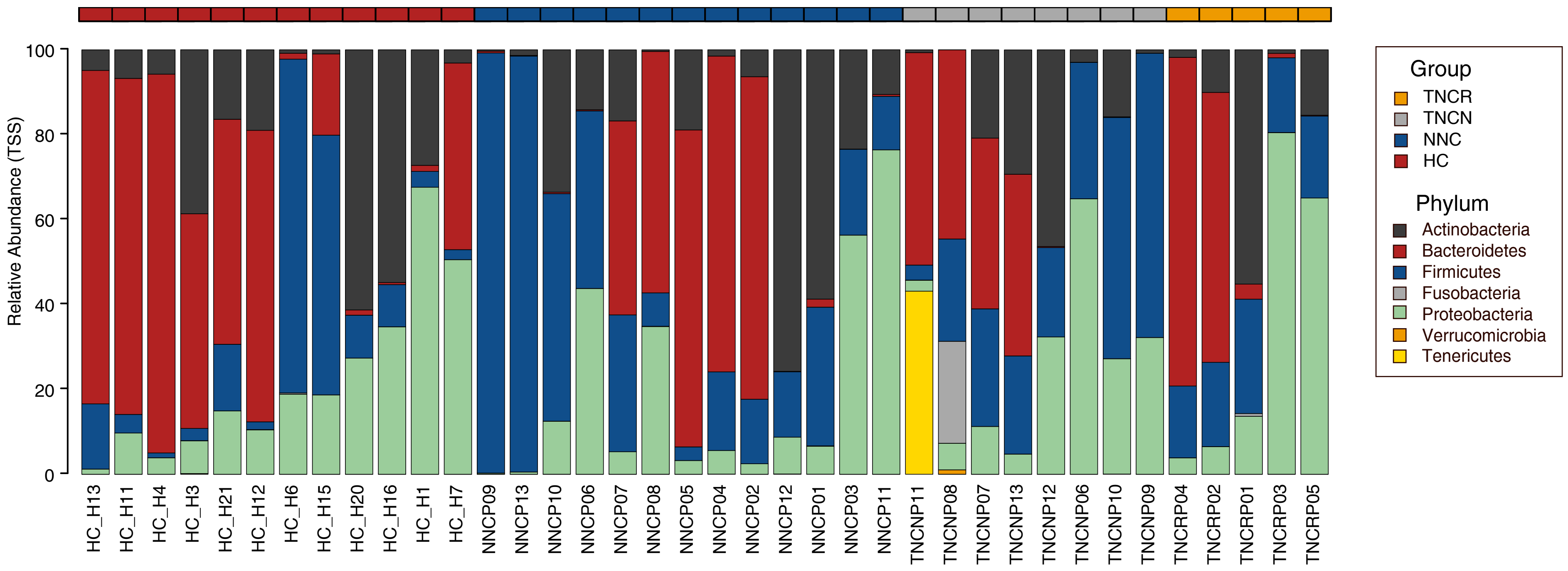

Additional file 2: microbiota distribution on phylum level. Yellow, darkred, gray, and blue represents TNCR, HC, TNCN, and NNC groups.

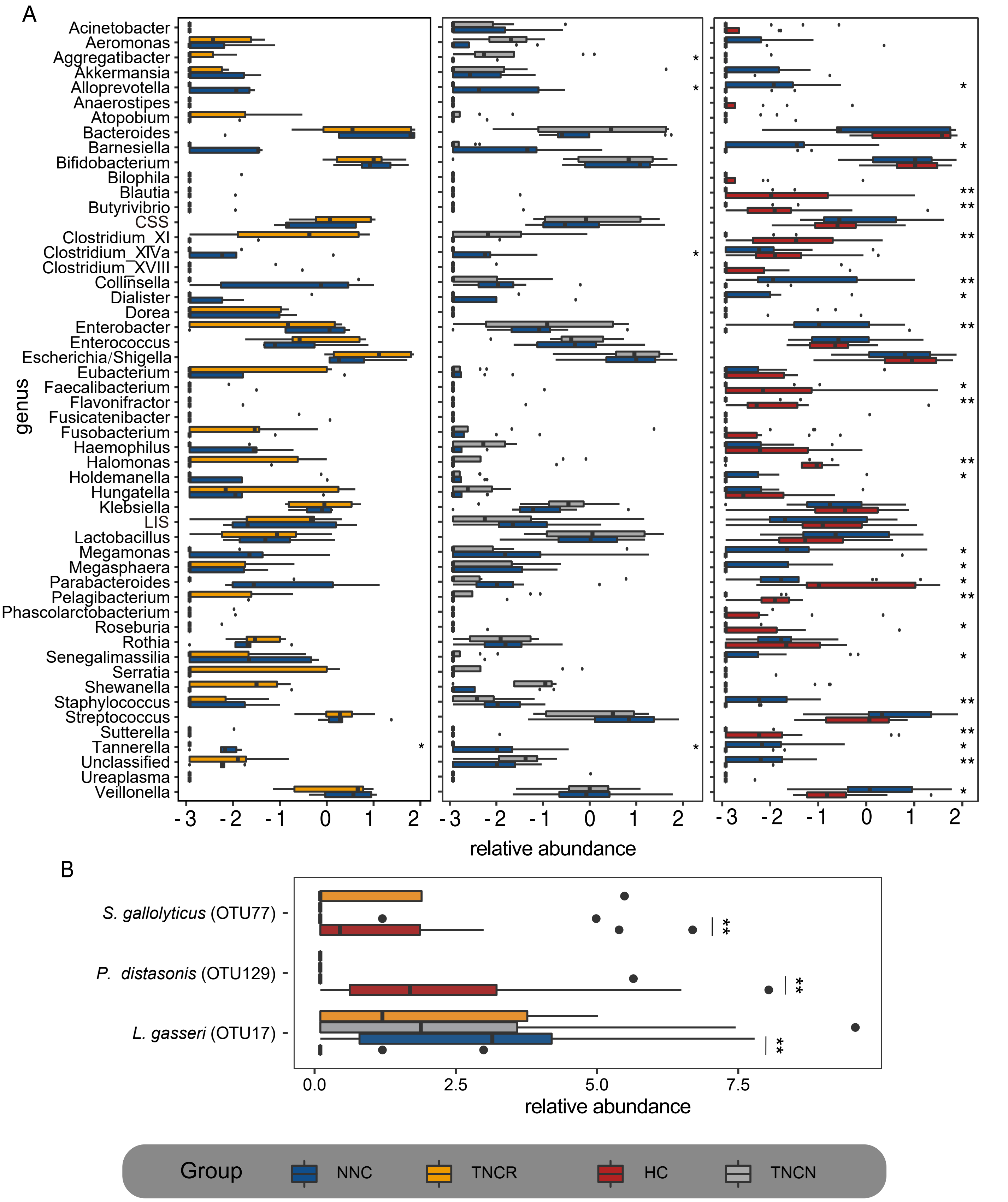

Additional file 3: microbiota difference analysis on genus level (A) and OTU level (B). *, *P*<0.05; **, *P*<0.01.

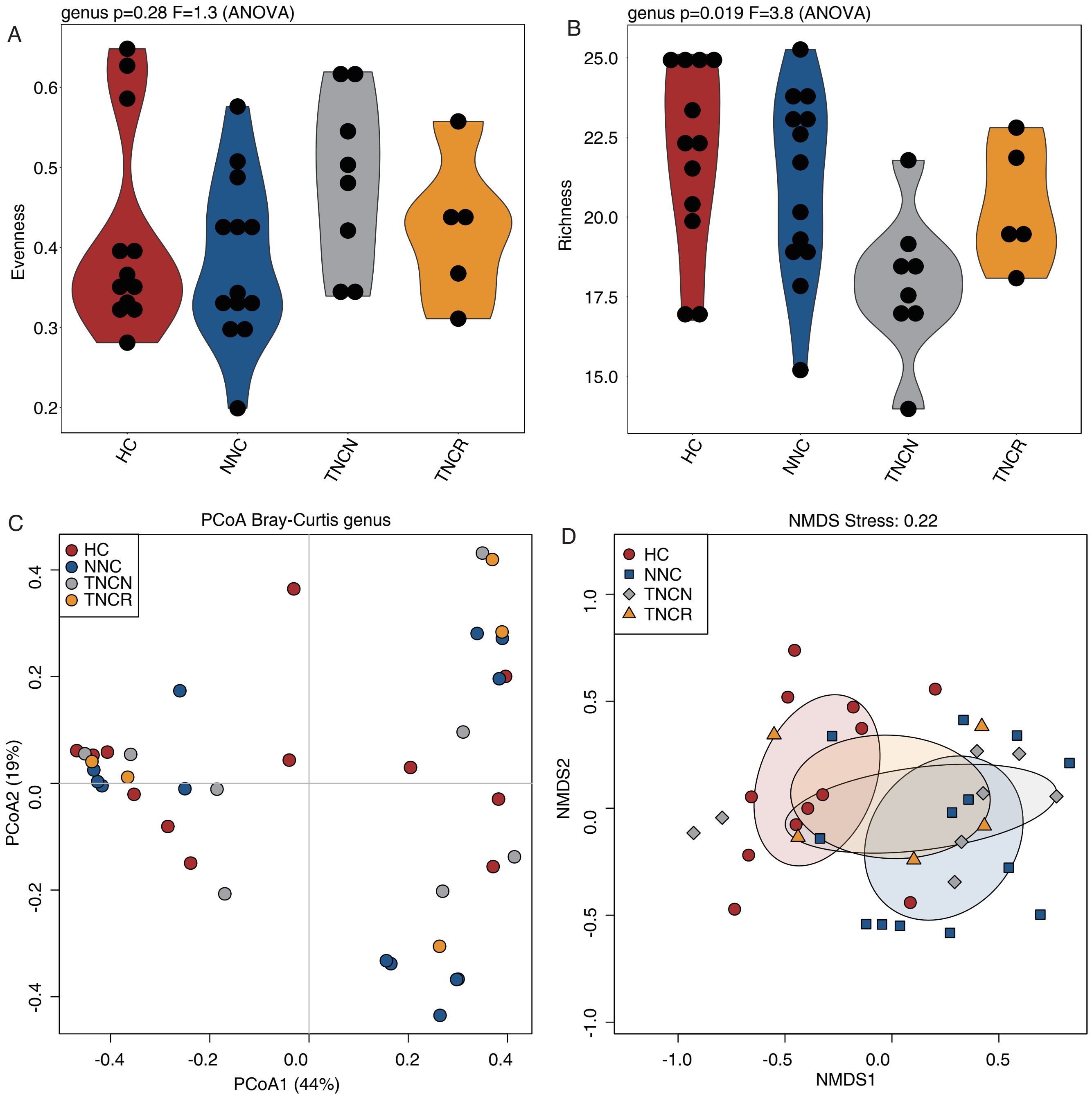

Additional file 4: part of microbiota alpha and beta analysis results. A and B were eveness and richness indexes of four groups. C and D shown microbiota beta diversity of four groups.
